# Gray Matter Abnormalities in Type 1 and Type 2 Diabetes: A Dual Disorder ALE Quantification

**DOI:** 10.3389/fnins.2021.638861

**Published:** 2021-06-07

**Authors:** Kevin K. K. Yu, Gladys L. Y. Cheing, Charlton Cheung, Georg S. Kranz, Alex Kwok-Kuen Cheung

**Affiliations:** ^1^Department of Rehabilitation Sciences, The Hong Kong Polytechnic University, Kowloon, Hong Kong; ^2^University Research Facility in Behavioral and Systems Neuroscience (UBSN), The Hong Kong Polytechnic University, Kowloon, Hong Kong; ^3^Department of Psychiatry, The University of Hong Kong, Pokfulam, Hong Kong; ^4^The State Key Laboratory for Brain and Cognitive Sciences, The University of Hong Kong, Pokfulam, Hong Kong; ^5^Department of Psychiatry and Psychotherapy, Medical University of Vienna, Vienna, Austria

**Keywords:** anatomical likelihood estimation, diabetes mellitus, voxel-based morphometry, meta-analysis, systematic review

## Abstract

**Aims/hypothesis:** Diabetes mellitus (DM) is associated with comorbid brain disorders. Neuroimaging studies in DM revealed neuronal degeneration in several cortical and subcortical brain regions. Previous studies indicate more pronounced brain alterations in type 2 diabetes mellitus (T2DM) than in type 1 diabetes mellitus (T1DM). However, a comparison of both types of DM in a single analysis has not been done so far. The aim of this meta-analysis was to conduct an unbiased objective investigation of neuroanatomical differences in DM by combining voxel-based morphometry (VBM) studies of T1DM and T2DM using dual disorder anatomical likelihood estimation (ALE) quantification.

**Methods:** PubMed, Web of Science and Medline were systematically searched for publications until June 15, 2020. VBM studies comparing gray matter volume (GMV) differences between DM patients and controls at the whole-brain level were included. Study coordinates were entered into the ALE meta-analysis to investigate the extent to which T1DM, T2DM, or both conditions contribute to gray matter volume differences compared to controls.

**Results:** Twenty studies (comprising of 1,175 patients matched with 1,013 controls) were included, with seven studies on GMV alterations in T1DM and 13 studies on GMV alterations in T2DM. ALE analysis revealed seven clusters of significantly lower GMV in T1DM and T2DM patients relative to controls across studies. Both DM subtypes showed GMV reductions in the left caudate, right superior temporal lobe, and left cuneus. Conversely, GMV reductions associated exclusively with T2DM (>99% contribution) were found in the left cingulate, right posterior lobe, right caudate and left occipital lobe. Meta-regression revealed no significant influence of study size, disease duration, and HbA1c values.

**Conclusions/interpretation:** Our findings suggest a more pronounced gray matter atrophy in T2DM compared to T1DM. The increased risk of microvascular or macrovascular complications, as well as the disease-specific pathology of T2DM may contribute to observed GMV reductions.

**Systematic Review Registration:** [PROSPERO], identifier [CRD42020142525].

## Introduction

Diabetes mellitus (DM) is a common disease affecting more than 451 million people worldwide, and its prevalence may increase to 693 million cases by 2,045 (Cho et al., 2018). DM is divided into two subtypes, type 1 diabetes (T1DM) and type 2 diabetes (T2DM). Both subtypes are associated with persistent hyperglycemia, but have distinct causes, a different age at onset and different pathophysiologies (Leslie et al., 2016). T1DM has an onset in childhood and young adulthood and is characterized by insulin deficiency due to an autoimmune attack of insulin producing pancreatic beta cells. Conversely, with its onset in adulthood, T2DM is a chronic condition characterized by the body's increasing inability to either respond to functional insulin effectively and/or produce sufficient insulin for normal glucose regulation. Because of impaired glucose metabolism, it is widely accepted that both types of DM share increased risk in similar clinical features and complications, primarily vascular disease such as retinopathy, neuropathy, nephropathy, and cardiovascular disease.

Growing attention has been paid to the effect of DM on central nervous system because proper glucose regulation is essential for optimal brain functioning. Cognitive decrement has been observed in neuropsychological tests among diabetic patients; in particular, information processing speed and psychomotor efficiency were more affected than other cognitive functioning domains by the disease (Ryan et al., 2003; Brands et al., 2006). Furthermore, DM has been found to be associated with increased risk of Alzheimer disease. Quantitative meta-analysis of longitudinal studies identified higher relative risk of Alzheimer disease of 1.5 (95% CI 1.2–1.8) and vascular dementia of 2.5 (95% CI 2.1–3.0) among diabetic patients when compared with their nondiabetic counterparts (Cheng et al., 2012). Collectively, both types of DM have been shown to be associated with reduced cognitive function. While several studies indicated more pronounced dysfunctions in T2DM compared to T1DM, direct comparisons showed no systematic differences in cognitive abilities such as abstract reasoning, memory, attention and executive function, visuoconstruction, and information processing speed (Brands et al., 2007).

Brain imaging such as magnetic resonance imaging (MRI) is an ideal means to explore the neural correlates of cognitive dysfunction in DM. Altered cerebral metabolism has been observed in T1DM and T2DM (Sarac et al., 2005; Sinha et al., 2014). In addition, structural neuroimaging revealed reduced gray matter volume (GMV) in both types of DM. However, results were inconsistent, which may be attributed to numerous variables including differences in sample size, imaging devices and protocols used (Gold et al., 2007; Chen et al., 2012; Moran et al., 2013; Zhang et al., 2014). Direct comparisons of MRI ratings of white matter lesions and cortical atrophy by Brands et al. (2007) revealed more pronounced deep white matter lesions and cortical atrophy in T2DM compared to T1DM (Brands et al., 2007). A more recent study by Moulton et al. (2015) attempted to review neuroimaging research including voxel-based morphometry (VBM) data and volumetric data using meta-analysis (Moulton et al., 2015). The authors performed separate meta-analyses for T1DM and T2DM and found reduced bilateral thalamus in T1DM whereas reduced global brain volume and regional atrophy in the hippocampi, basal ganglia, and orbitofrontal and occipital lobes were seen in T2DM. However, a comparison of VBM data of both types of DM in a single analysis has not been done so far. Yet, such an analysis would be needed in order to investigate the distinctiveness or similarities of T1DM and T2DM directly in an unbiased objective comparison.

VBM is an automated whole-brain based analysis method that has several advantages over a region-of-interest (ROI)-based approach. VBM measures local volume or concentration of gray matter voxel-wise across the whole brain. Thus, in order to conduct an unbiased objective investigation of neuroanatomical differences in DM, the aim of this study was to conduct a meta-analysis combining VBM studies of T1DM and T2DM using the anatomical likelihood estimation (ALE) technique.

## Methods

### Literature Search

Our meta-analysis was registered with PROSPERO (registration number CRD42020142525) and was conducted according to the Preferred Reporting Items for Systematic Reviews and Meta-Analyses (PRISMA) guidelines (Moher et al., 2009). The studies were selected from PubMed (https://pubmed.ncbi.nlm.nih.gov/), Web of Science (https://www.webofknowledge.com/) and Google Scholar (https://scholar.google.com.hk/) databases and were limited to publications before October 1, 2020. The keywords used were “diabetes” or “diabetes mellitus” or “DM” plus “VBM,” “voxel-based,” “voxel-wise,” “morphometry,” or “VBM.” In addition, review articles and reference lists of identified articles were manually checked. Individual articles had to meet the following inclusion criteria:

1. Gray matter differences between patients with DM and non-DM controls were compared
2. Comparison was performed at the whole-brain level
3. The gray matter differences between patients and controls were reported in a stereotactic space in three coordinates (x, y, z), either in Montreal Neurological Institute (MNI) or Talairach space.
4. Coordinates were included as separate studies if they contained multiple independent patient samples.
5. Studies using ROI or seed voxel-based analysis were excluded.
6. For studies lacking the Talairach or MNI coordinates, study authors were contacted in order to minimize the possibility of a biased sample set.
7. Studies considered for inclusion had to be published in English in a peer-reviewed journal
8. Subjects included had to have formal diagnosis of either type 1 or type 2 diabetes. Moreover, voxel-based imaging methods and co-ordinates reported in 3D stereotactic space had to be used.

Studies restricted to males/females or children/adults were included. Studies presenting overlapping or identical samples were identified, and only the study presenting the largest number of subjects was retained. If there was possible overlapping but different results were presented, e.g., hippocampus presented in one study while frontal lobe in another, all data were included.

### Quality Assessment

A customized checklist was used to assess the quality of included studies, as done by others (Katon et al., 2010) (Table 1). The checklist contained 12 items, and was based on previous meta-analytic studies (Shepherd et al., 2012; Du et al., 2014) with additional parameters including the diagnostic procedures, the demographic and clinical characterization, the sample size, the MRI acquisition parameters, the analysis technique and the quality of the reported results. Due to the rapid changing of data-processing methods, we included a new item “included modern MRI processing methods of past 10 years” in the checklist (item 8). The checklist provided objective information about the quality of included studies. Each study was reviewed by two authors (K.K.K.Y, G.S.K), and a completeness rating was independently determined. If ratings disagreements arose, the papers were discussed, after which a consensus score was obtained. Only studies with quality score of 8 or above were included in the analysis.

**Table 1:** Customized checklist for study quality assessment (adopted from Du et al., 2014).

	**Category 1: Subjects**
1	Patients were evaluated prospectively, specific diagnostic criteria were applied, and demographic data was reported
2	Healthy comparison subjects were evaluated prospectively, psychiatric and medical illnesses were excluded and demographic data was reported
3	Important variables (e.g., age, gender, intelligence quotient, i.e., IQ, handedness, socio-economic status, height, or total brain measures) were checked, either by stratification or statistically
4	Sample size per group > 10
	**Category 2: Methods for image acquisition and analysis**
5	Magnet strength at least 1.5 T
6	MRI slice-thickness ≤ 3 mm
7	Whole brain analysis was automated with no a priori regional selection
8	Modern MRI processing methods of past 10 years
9	The imaging technique used was clearly described so that it could be reproduced
10	Measurements were clearly described so that they could be reproduced
	**Category 3: Results and conclusions**
11	Statistical parameters for significant, and important non-significant, differences were provided
12	Conclusions were consistent with the results obtained and the limitations were discussed

### ALE Procedure

ALE treats each foci reported in VBM as a probability distribution in order to test for agreement across studies (Turkeltaub et al., 2002; Laird et al., 2005; Ellison-Wright et al., 2008). Typically, ALE is applied on a single disorder to identify volumetric differences consistently reported across VBM studies. The result of this approach is an ALE map showing the same regions that are consistently reported across studies. In the present study, we adopted the “Dual Disorder ALE Quantification.” We have previously applied this method to study similarities across different disorders such as schizophrenia and bipolar disorder, and schizophrenia and autism (Cheung et al., 2010; Yu et al., 2010; McAlonan et al., 2011). In brief, a map of gray matter difference compared to controls was generated for each study. These “gray matter difference” maps were categorized based on their disorder type, and averaged into a mean map. As a result, a mean map of T1DM and a mean map of T2DM were created. The mean maps were combined to form a total gray matter difference map, after which whole brain permutation testing (Turkeltaub et al., 2002), and controlled false discovery rate (FDR) thresholding was conducted (Laird et al., 2005). These procedures were conducted using an ALE kernel (Leung et al., 2009) available from the open source software available at http://csl.georgetown.edu/software/ (Turkeltaub et al., 2002). The intensity of the mean disorder maps and the intensity of the final ALE result were divided such that the intensity ratio for each resultant cluster was calculated (ALE kernel and customized scripts for Matlab and SPM12).

The first stage of ALE is to generate a Gaussian distribution surrounding the central coordinates for each significant focus reported in studies. The probability that any given voxel is linked to the disorder(s) in question can be quantitatively estimated from this whole brain likelihood map. ALE eliminates unlikely foci and only points to likely foci that are close in proximity, in effect outlining regions which were reported most often across studies, to generate resultant three-dimensional clusters. It is emphasized that the approach of this study was to combine datasets from both disorders into the same entry for a single analysis. In order to do so, individual “likelihood” maps that reflect the probability of finding gray matter differences, were generated for each of the included studies. A study with no findings across subjects and controls were represented by an empty map. Each of the likelihood maps were grouped based on the type of DM (T1 or T2), and averaged together into a mean likelihood map of conditions. The purpose for generating the mean maps was to avoid bias toward the condition with more reported foci. The mean maps were summated together to a joint likelihood map and 10,000 permutations were used to sample the null distribution. The result was thresholded by FDR (*p* < 0.05) and clusters smaller than 100 mm^3^ were filtered. The resultant ALE map then contained clusters consisting of foci from T1DM, or T2DM, or both conditions. The contribution of each disorder to every resultant cluster was calculable. Two separate ALE analyses were performed for reductions and elevated gray matter volumes.

Finally, for each of the included studies, a “gray matter difference” map was generated to determine how much each study contributes toward the resultant ALE clusters. This contribution score was then used for meta-regression to test whether demographics or clinical measures including the study size, disease duration, and % glycated hemoglobin (HbA1c) have any influences toward the ALE result.

## Results

### Studies Demographics

**Figure 1:**
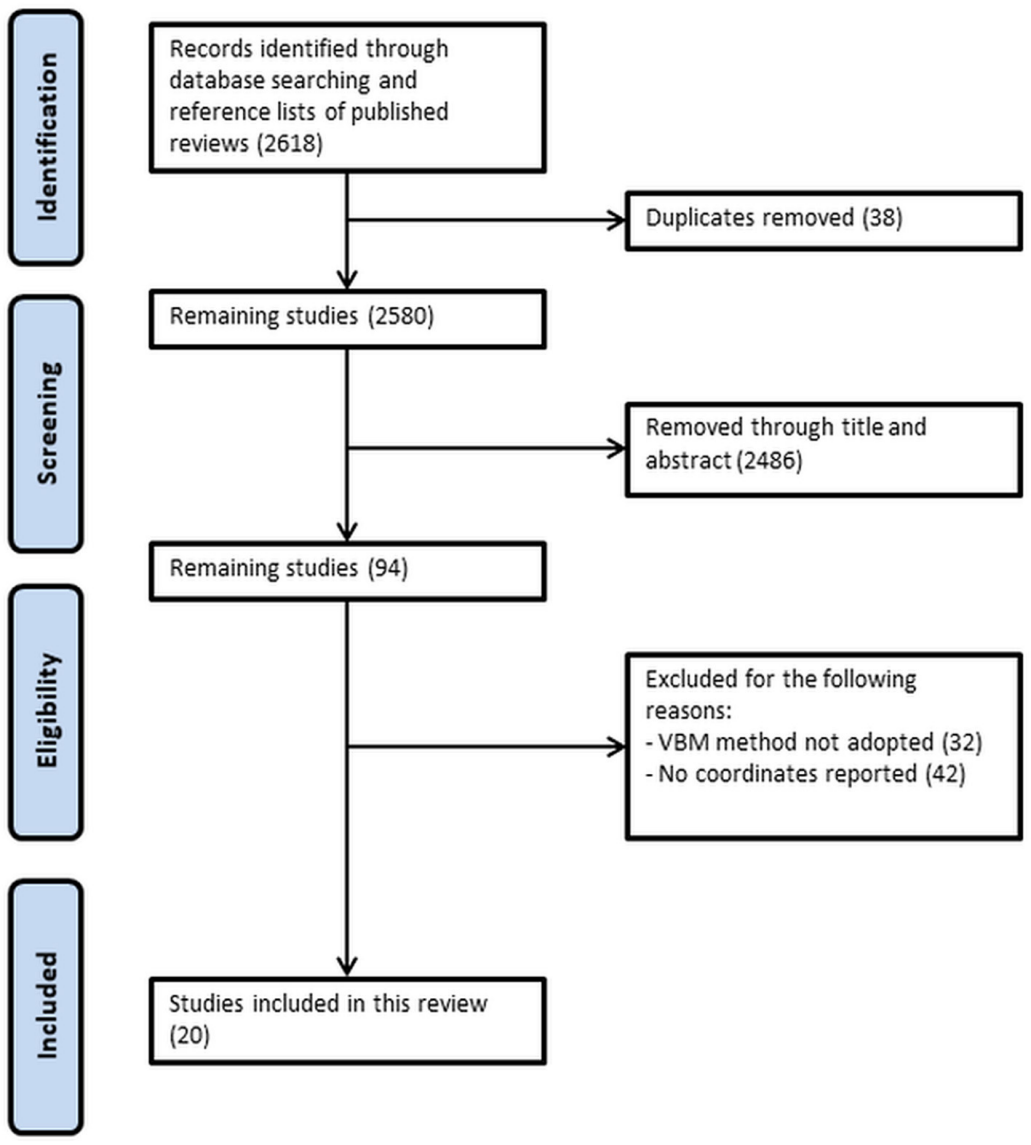
Preferred Reporting Items for Systematic Reviews and Meta-analyses (PRISMA) flow diagram of search strategy.

Figure 1 shows the detailed selection process of included studies. After screening through title and abstract and removal of duplicates, a total of 94 studies were checked for eligibility. Among which, 32 studies were excluded as the VBM method was not adopted, and 42 studies were not included because the coordinates representing gray matter differences were not reported. A total of 20 studies were included in this analysis, with seven studies and 13 studies describing gray matter alterations in T1DM and T2DM respectively (see Table 2). A total of 1,175 patients matched with 1,013 controls were included. The T1DM group was significantly younger than the T2DM group (with a mean age of 23.7 compared to 49.8 years, respectively). However, there were no significant differences in age and sex between patient groups and their respective control groups. A total of 509 patients in the T1DM group were matched with 351 controls, whereas a total of 666 patients constituted the T2DM group that was matched with 662 control participants. T1DM had diabetes for an average of 14.7 years which was double than the average 7.3 years of T2DM, although this difference did not reach significance given the considerable variance between studies (see Table 2). Both groups had comparable HbA1c levels (T1DM: 8.6; T2DM: 8.3).

**Table 2:** Papers included in the current meta-analysis.

**References**	**Number of patients**	**Number of controls**	**Mean age of patients**	**Mean age of controls**	**Diabetes type**	**Diabetes duration (years)**	**HbA1c (%)^**#**^**	**Number receiving anti-diabetic therapy**	**MRI preprocessing methods**	**Quality score**
Kaufmann et al. (2012)	30	19	14.3 ± 4.0	13 ± 3.2	T1DM	5.6 ± 3.8	8.4 ± 0.9	30	SPM	9.5
Liu et al. (2019)	21	21	9.3 ± 2.1	9.4 ± 1.1	T1DM	0.6 ± 0.1	11.2 ± 2.2	21	FSL	9
Marzelli et al. (2014)	142	68	7.0 ± 1.7	7 ± 1.8	T1DM	2.9 ± 2.0	7.9 ± 0.9	142	SPM	10
Musen et al. (2006)	82	36	32.6 ± 3.2	31.3± 5.1	T1DM	20.3 ± 3.6	7.8 ± 1.3	82	Analyze	10
Nunley et al. (2017)	95	135	49.1 ± 6.7	48.7 ± 7.3	T1DM	40.9 ± 6.2	n/a	95	FSL	10
Perantie et al. (2007)	108	51	12.6 ± 2.7	12.3 ± 2.7	T1DM	5.7 ± 2.9	8.4 ± 1.0	108	SPM	10
Wessels et al. (2006)	31	21	40.8 ± 5.9	36.3 ± 7.9	T1DM	26.8 ± 8.3	8.0 ± 1.1	31	SPM	10
	**509**	**351**	**23.7** **±** **3.8**	**22.6** **±** **4.2**		**14.7** **±** **3.8**	**8.6** **±** **1.2**	**509**		
Chen et al. (2012)	16	16	61.2 ± 7.8	59.6 ± 6.1	T2DM	13.2 ± 5.6	8.4 ± 1.7	n/a	SPM	10
Chen et al. (2017)	23	24	60.8 ± 8.3	57.0 ± 7.5	T2DM	9.0 ± 4.8	8.6 ± 2.2	12	SPM	10
Cui et al. (2017)	40	41	60.5 ± 6.9	57.9 ± 6.5	T2DM	8.9 ± 5.0	7.7 ± 1.6	8	SPM	9
Fang et al. (2019)	35	32	32.1 ± 5.3	34.1 ± 4.8	T2DM	1	10.4 ± 2.4	33	SPM	11
Ferreira et al. (2017)	24	27	58.6 ± 8.6	59.9 ± 5.9	T2DM	8.0 ± 7.9	10.0 ± 2.8	n/a	SPM	8.5
García-Casares et al. (2014)	25	25	60.0 ± 4.6	57.8 ± 5.4	T2DM	11.25 ± 7.9	6.7 ± 0.8	25	SPM	10
Moran et al. (2013)	350	363	67.8 ± 6.9	72.1± 7.2	T2DM	7 (median)	7.2 ± 1.2	72	SPM	10
Nouwen et al. (2017)	14	19	16.1 ± 1.5	16.4 ± 1.7	T2DM	2.7 ± 2.5	8.1 ± 2.3	12	SPM	10
Redel et al. (2018)	20	20	16.7 ± 2.0	16.7 ± 2.6	T2DM	2.8 ± 2.1	7.9 ± 2.2	18	SPM	9
Wang et al. (2014)	23	23	53.1 ± 9.6	53.9 ± 9.2	T2DM	7	8.3 ± 1.4	n/a	SPM	9.5
Wang et al. (2017)	17	17	54.8 ± 8.3	54.4 ± 7.9	T2DM	n/a	n/a	n/a	SPM	12
Zhang et al. (2014)	53	29	54.2 ± 8.5	55.48	T2DM	7.3 ± 5.7	7.6 ± 1.5	n/a	SPM	10
Zhang et al. (2019)	26	26	51.9 ± 10.7	48.2 ± 6.7	T2DM	9.2 ± 7.1	n/a	21	SPM	10
	**666**	**662**	**49.8** **±** 6.8^**^	**49.5** **±** 6.0^**^		**7.3** **±** **5.4**^**ns**^	**8.3** **±** **1.8**^**ns**^	**201**		

*Values represent mean ± SD if not stated otherwise*.

*Bold rows depict sum scores for number of patients, number of controls and number receiving anti-diabetic therapy, and average scores for mean age of patients and controls, diabetes duration and HbA1c values*.

** *Indicates a significant difference (p < 0.01) between T1DM and T2DM*.

*^ns^Indicates no significant difference between T1DM and T2DM (independent samples T-test)*.

*T1DM, type-1 diabetes mellitus; T2DM, type-2 diabetes mellitus; HbA1c (%), Hemoglobin A1C (^#^provided for the DM group)*.

### Results of Gray Matter Alterations From ALE

**Figure 2:**
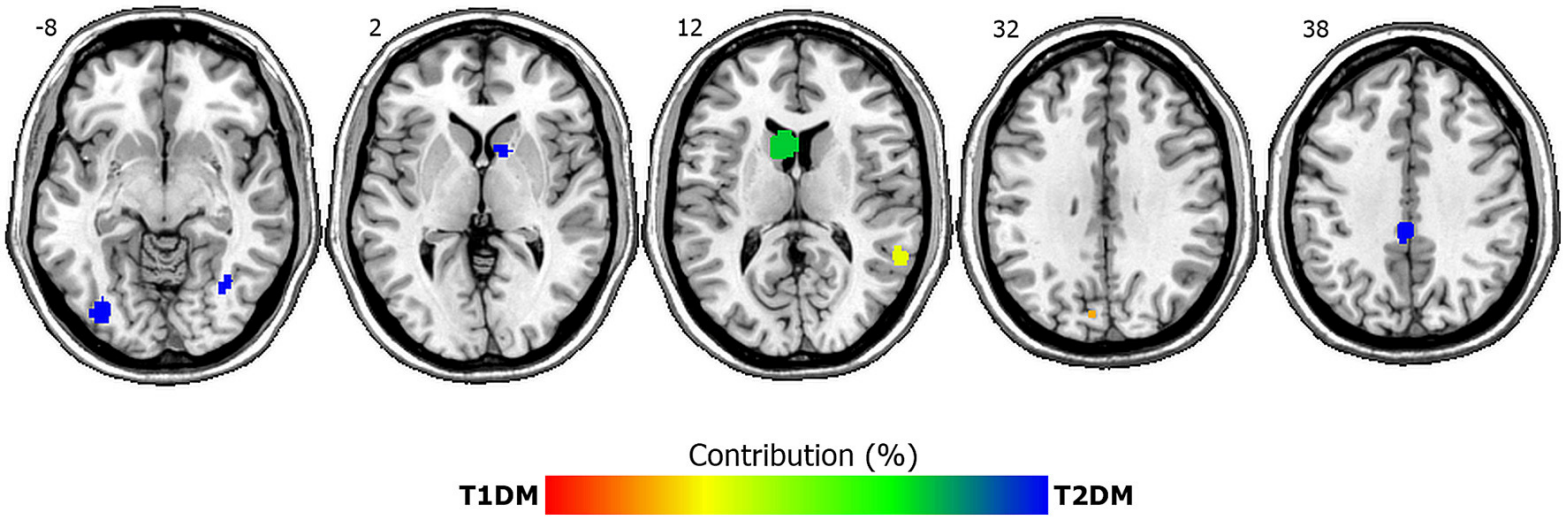
ALE clusters of lower gray matter volumes in T1DM and T2DM compared to controls. Depicted are seven significant clusters, overlaid onto axial structural MRI planes Numbers depict z-coordinates in MNI space. Blue clusters are driven by T2DM whereas both disorders contribute to green-yellow clusters. Percent contribution is signaled by the color bar, with red indicating 100% contribution by T1DM and blue indicating 100% contribution by T2DM. Left is left.

ALE analysis for GMV reductions revealed seven clusters of lower GMV in T1DM and T2DM patients relative to controls across studies (see Table 3 and Figure 2). Both DM subtypes showed GMV reductions in the left caudate, right middle temporal lobe and left cuneus (BA 19). Whereas reductions in left cuneus and right middle temporal lobe were more driven by T1DM, left caudate reductions were stronger in T2DM. Conversely, GMV reductions associated exclusively with T2DM (>99% contribution) were found in the left cingulate (BA 31), right inferior temporal lobe, right caudate and left occipital lobe. GMV reductions associated mainly with T1DM were not present (for the exact % of contribution for each cluster, see Table 2). The ALE analysis for GMV increases revealed no significant clusters for any DM subtype.

**Table 3:** ALE clusters of lower gray matter volumes in T1DM and T2DM compared to controls.

**Cluster**	**MNI coordinates**	**Location**	**T1DM %**	**T2DM %**
1	(−1, −31, 41)	Left cingulate (BA 31)	0	100
2	(39, −67, −4)	Right inferior temporal lobe	0	100
3	(14, 12, −3)	Right caudate	0.01	99.99
4	(−37, −84, −3)	Left occipital lobe	0.02	99.98
5	(−7, 17, 9)	Left caudate	24.22	75.78
6	(64, −49, 15)	Right middle temporal lobe	64.76	35.24
7	(−6, −81, 42)	Left cuneus (BA 19)	78.41	21.59

Finally, a meta-regression to investigate the potential influence of study size (number of included participants), disease duration, and % glycated hemoglobin (HbA1c) revealed no significant influence of these covariates, neither when tested individually, nor when combined in one regression model.

## Discussion

This ALE meta-analysis made use of 20 VBM studies including seven studies of T1DM patients and 13 studies of T2DM patients to reveal overlaps and differences in GMV alterations between both conditions. Our analysis showed only GMV reductions in diabetic patients compared to controls, but no GMV increases. At first glance, this is not surprising given that hyperglycemia leads to cellular damage, as seen in rodent studies (Sadeghi et al., 2016; Hamed, 2017). More specifically, our results can be explained by insulin resistance, the principal characteristics in DM, which lowers glucose metabolism in the brain, resulting in enhanced amount of plasma glucose in DM patients (Baker et al., 2011). Chronic hyperglycemia is a potential determinant for diabetes-induced problems in the brain, as it could cause metabolic and molecular alterations, leading to neuron dysfunction or death in the brain (Tomlinson and Gardiner, 2008). Similar to Alzheimer's disease (AD), tau phosphorylation and activation of advanced glycation end products (AGE) have been known to contribute to multiple proinflammatory cytokine release that eventually leads to synapse reduction and neuronal loss in diabetic brain (Zhao et al., 2018). Consequently, the resulting neuronal loss and gray matter atrophy that accounted for the frequently observed cognitive dysfunctions in DM are observable by brain MRI as GMV reductions (Brands et al., 2005).

Our meta-analysis shows a preponderance of GMV reductions in T2DM compared to T1DM, although T2DM appears to be better as compared to that of T1DM from the perspective of disease duration of patients as well as the glycemic control. Our meta-regression analysis revealed no influence of study size, disease duration, or HbA1c values on GMV, further suggesting that the involvement of other contributing factors to the GMV reductions. It is, in fact, not entirely out of our expectation considering that the etiologies of the two types are quite different. A recent review from Tamarai et al. (2019) have indicated obvious differences in the known genetic variants associated with the two types of DM (Tamarai et al., 2019). T1DM is a result of insufficient insulin secreting β-cells, and the genetic variations associated with T1DM are mainly related to alterations in insulin synthesis. While T2DM demonstrates impaired mechanisms of insulin release in response to hyperglycemia in addition to β-cells deficiency. The complexity of T2DM pathophysiology can be comprehended by interaction between multiple genes scattered all across the genome, as well as the interaction between genetic factors and environmental factors (e.g., life style; Tamarai et al., 2019).

Lower GMV in T2DM compared to T1DM is in accordance with a comparative study by Brands et al. (2007) who showed that MRI ratings of cortical atrophy are worse in T2DM compared to T1DM (Brands et al., 2007). Microvascular or macrovascular complications and comorbidities are more likely in T2DM than T1DM even when investigating youth-onset DM and adjusting for age (Luk et al., 2014; Dabelea et al., 2017). While it is possible that comorbid conditions, such as hypoglycaemia, hypercholestrolaemia, and hypertension may explain the difference, cognitive dysfunctions may also explain the differences in GMV reductions between DM types. Cognitive impairments seem to be stronger in T2DM compared to T1DM (Awad et al., 2004; Brands et al., 2005; Zilliox et al., 2016), but a direct comparison of the two DM types revealed no significant differences in cognitive dysfunctions (Brands et al., 2007).

GMV reductions in our study were confined to seven clusters in specified brain regions including left and right caudate, temporal, occipital lobes, and cingulate cortex. GMV reductions in caudate, cingulate, inferior temporal, occipital lobe were exclusively driven by T2DM. These results partly concur with a volumetric meta-analysis by Moulton et al. (2015) who also observed occipital and caudate GMV reductions in T2DM (Moulton et al., 2015). The caudate exhibits a high insulin receptor density, so GMV in this region may be especially vulnerable to diabetes-associated atrophy (Schulingkamp et al., 2000). Interestingly, a recent transcriptomic analysis conducted with over 300 T2DM samples found that the T2DM-associated genes are expressed in the caudate significantly more than other brain regions (Zhou et al., 2019). Functional analysis revealed that these T2DM-associated genes affects synaptic functions and are related to other neurodegenerative diseases.

Co-occurrence of DM and depression was observed previously (Katon et al., 2010; Balhara, 2011; Roy and Lloyd, 2012; Bãdescu et al., 2016), and the prevalence of developing depression is three times higher in T1DM patients and two times higher in T2DM patients as compared to general population (Roy and Lloyd, 2012). Moreover, those with depression are 60% more likely to develop T2DM (Mezuk et al., 2008). Consistent with our result, in structural and functional connectivity studies of depression disorder, it was reported that there is lower gray matter in the bilateral caudates (Shah et al., 2002; Kim et al., 2008; Ma et al., 2012) and right middle temporal gyrus (Peng et al., 2011; Ma et al., 2012; Kandilarova et al., 2019), and altered functional connectivity in the right caudate and right middle temporal gyrus (Ma et al., 2012). Deficits of these regions may suggest shared pathways that contribute to DM and depression.

GMV reductions in the cingulate cortex observed in our study were confined to a cluster in the posterior cingulate cortex (PCC). The PCC is considered as one of the “key hub” of the DMN, and is associated with functions such as memory retrieval (Gusnard et al., 2001a) and regulating attention (Gusnard et al., 2001b). It has been reported that T2DM subjects have poorer memory and attention impairments as compared to matched controls (Gregg et al., 2000; Kanaya et al., 2004; van den Berg et al., 2010). Also, resting-states fMRI meta-analysis using ALE demonstrates that the PCC is affected in T2DM patients (Xia et al., 2017). Other quantitative fMRI studies using functional connectivity also show that resting-states is altered in the PCC (Cui et al., 2015; Ishibashi et al., 2018). These studies suggest that T2DM may have a disrupted DMN. Furthermore, Chen et al. found reduced functional activity in the PCC in T2DM patients when performing an encoding task related episodic memory, suggesting that DMN is affected in T2DM (Chen et al., 2016). It has also been observed that fractional anisotropy (FA) of the cingulum bundle are correlated to PCC and the medial frontal gyrus, which are important regions of the DMN (Hoogenboom et al., 2014). A previous meta-analysis speculated that gray matter volume differences in the DMN regions including PCC may be the reason why brain activation is affected in the DMN of T2DM patients, in terms of functional connectivity and activity, and ultimately leading to reduced cognitive performance (Liu et al., 2017).

DM (especially T2DM) and AD both shared some common neurocognitive functional deficits, one of which is the impaired memory (Karvani et al., 2019; Backeström et al., 2021). Most research, especially using animal model, places hippocampus as the center of focus on memory loss. While hippocampal atrophy has been observed in T2DM, enlarged hippocampus was reported in T1DM (Hershey et al., 2010; Heyden et al., 2011), further indicating the mechanistic differences between T1DM and T2DM. Hippocampus, located deep within the temporal lobe, is not the only region responsible for memory function. Middle and Inferior temporal gyri, which are relatively superficial as compared to hippocampus, also play critical role in memory. Our data has revealed GMV reductions in right middle temporal gyrus and right inferior temporal gyrus. Middle and inferior temporal gyri (Musen et al., 2006; Chen et al., 2012; Wang et al., 2014; Redel et al., 2018; Zhang et al., 2019) have been associated with semantic memory and semantic priming, in which semantically related stimuli resulted in faster or more effective activation. Early study has already shown a reduced cerebral blood flow in temporal lobe (Jimenez-Bonilla et al., 1996), which is believed to induce neuronal cell loss that resulted in temporal gyri atrophy that accounts for the reduced GMV of the respective regions.

Our data also indicated that left occipital lobe, and left cuneus which is also located in occipital lobe, demonstrated differential GMV in T2DM as compared to control. Occipital lobe is the center for visual processing, and it is possible that differential GMV could be a consequence of early sign of diabetic retinopathy. For example, glaucoma induced retinal damages has been shown to correlate with atrophy in occipital lobe, in particular the BA19 (Jiang et al., 2017). BA19 is located in parts of the cuneus and lingual gyrus. While lingual gyrus is associated with visual memory, cuneus is known to relate to inhibitory control (Haldane et al., 2008; Wang et al., 2018), the ability to inhibit or control impulsive responses by using attention and reasoning. Dysfunction in inhibition, although best known in people with attention deficits and hyperactivity disorder (ADHD), is also observed in T2DM (Cooke et al., 2020). In addition, strong correlation was observed between impaired cognitive performance in T2DM patients and reduced blood flow in cerebral regions, one of which was the occipital lobe (Cui et al., 2017). Therefore, GMV reduction in occipital lobe and cuneus may represent not only visual but also cognitive deficits.

Schizophrenia has long been found to link with increased risk of T2DM, as the prevalence of type 2 diabetes is 2–5-fold higher in patients with schizophrenia when compared with those without DM (Mamakou et al., 2018). While this may due to the impact of antipsychotic treatment and also the disease progression, the fact that drug native patients of schizophrenia were still at 1.27–1.63-fold of risk of having T2DM than general population (Cohen and De Hert, 2011) may suggested that there is uniquely shared risk factor between the two diseases. A review of the genetic databases found 37 common susceptibility genes between schizophrenia and T2DM (Mamakou et al., 2018). Association studies of the TCF7L2 gene in diabetes suggested increased risk of schizophrenia (Hansen et al., 2011; Alkelai et al., 2012).

Contrast to the suggested linkage between T2DM and schizophrenia, a large population study of over 800 k individuals in Finland suggested the reverse between T1DM and schizophrenia (Juvonen et al., 2007). The study found an incidence of 0.21/10,000 schizophrenia in type 1 diabetes, while it was 0.56 /1,000 schizophrenia in the general public, an over 60% reduction in risk of schizophrenia in type 1 diabetes. Our findings in predominantly larger contribution of T2DM in bilateral caudate deficit in gray matter echoes with the contradictive difference in linkage between T1DM and T2DM with schizophrenia. Bilateral caudate deficit was found in drug naïve patients of schizophrenia (Chua et al., 2007) but not with treated patients (Leung et al., 2009), suggesting caudate's role in the early stage and also in the treatment stage of schizophrenia.

In addition to focus given to the contribution of diabetes on cognitive dysfunction, association of antidiabetic treatment on cognitive performance on diabetic patients has also gained attention. A recent meta-analysis (Zhang et al., 2020) summarized 10 studies comprising 254,679 participants to determine the relationship between metformin therapy and cognitive function in T2DM patients, and compared metformin treatment with other antidiabetic drugs, including sulfonylureas, thiazolidinediones, and insulin. Despite all the treatments targeting T2DM, only metformin exhibited significant improvement in cognitive dysfunction, while insulin, suprisingly, aggravated cognitive dysfunction. Furthermore, such improvement was only significant in Americans and Europeans but not in Asian patients, indicating perhaps glycemic control alone might not be as effective in improving DM-induced cognitive dysfunction as expected. In addition to its primary antidiabetic action on reduction of glucose production in liver, metformin has also been shown to prevent neuronal cell death (El-Mir et al., 2008) and inhibited the molecular and pathological development of AD in cell culture model (Gupta et al., 2011). Metformin has been demonstrated to improve cognitive performance in AD patients (Cao et al., 2018) as well as in SAMP8 mice, one of the commonly used animal AD model, without altering blood glucose level (Farr et al., 2019), suggesting that this antidiabetic drug may improve cognitive function by acting on pathways other than glycemic control but the exact mechanism remained unclear. Although cognitive impairment in DM may arise from hyperglycemia, it is believed that a combinatorial effect of inflammation, oxidative stress, impaired cerebrovasculature, increase β-amyloid deposition, cerebral insulin resistance and formation of AGE all contribute to the progressive development of cognitive dysfunction in DM patients.

We acknowledge that there are a number of limitations to this study. First is the “file-drawer” problem which means that studies reporting null results are under-represented in the literature. This is a problem which all meta-analyses suffer. In this study, we tried to minimize this error by generating an empty ALE map for studies that reported no gray matter differences between patient groups and controls. However, such studies demonstrating no differences are uncommon and not likely to be published. Second, MRI methodology is continually being improved, and the data extracted from various studies were pre-processed and analyzed in different ways. It is unfortunate that there were not enough studies to control for confounding factors including modulation and smoothing. To reduce the difference in methodologies affecting the outcome of our present study, we made use of a customized checklist to assess the quality of each study. The quality scores (mean: 9.9; s.d: 0.7) provide an overview of rigorous of each study. Without checking for quality scores, it is possible that lower quality studies (ex: outdated MRI acquisition or data processing methods, and low sample size) could influence the results. Lastly, while all T1DM patients were medicated, only about one third of T2DM patients received medication, hence we cannot rule out that our results could partly reflect an effect of medication.

## Conclusions

Our meta-analysis using the ALE methodology indicated GMV reductions in seven brain regions in T1DM and T2DM relative to controls. Clusters of lower GMV associated with both diabetes types were found in left caudate, right middle temporal lobe and left cuneus, whereas clusters exclusively found in T2DM were located in left cingulate, right inferior temporal lobe, right caudate and left occipital lobe. Our results indicate a more pronounced gray matter atrophy in T2DM compared to T1DM. We interpret this finding in terms of microvascular or macrovascular complications and disease-specific pathology of T2DM. To our knowledge, this study is the first meta-analysis of VBM studies in patients with DM which highlights overlapping and distinct brain atrophy found in T1DM and T2DM. The results of our study will aid understanding of the underlying neurodegenerative process in T1DM and T2DM.

## Data Availability Statement

The original contributions presented in the study are included in the article/supplementary material, further inquiries can be directed to the corresponding author/s.

## Author Contributions

GC, KY, and AC designed and conceptualized the study. KY and CC performed the data analysis. GK interpreted the data. KY, GK, CC, and AC contributed to discussion. GK and KY wrote the manuscript. All authors edited and reviewed the manuscript and agreed to its final version.

## Conflict of Interest

The authors declare that the research was conducted in the absence of any commercial or financial relationships that could be construed as a potential conflict of interest.
